# The PEP++ study protocol: a cluster-randomised controlled trial on the effectiveness of an enhanced regimen of post-exposure prophylaxis for close contacts of persons affected by leprosy to prevent disease transmission

**DOI:** 10.1186/s12879-024-09125-2

**Published:** 2024-02-20

**Authors:** Duane C. Hinders, Anneke T. Taal, Suchitra Lisam, Aymée M. da Rocha, Nand Lal Banstola, Prativa Bhandari, Abhijit Saha, Jugal Kishore, Virginia O. Fernandes, Abu Sufian Chowdhury, Anna T. van ‘t Noordende, Liesbeth Mieras, Jan Hendrik Richardus, Wim H. van Brakel

**Affiliations:** 1 NLR, Amsterdam, The Netherlands; 2 NLR India, Delhi, India; 3 NHR Brasil, Fortaleza, Brazil; 4 NLR Nepal, Kathmandu, Nepal; 5 TLMI Bangladesh, Dhaka, Bangladesh; 6 Vardhman Mahavir Medical College/Safdarjung Hospital, Delhi, India; 7 Federal University of Ceará, Fortaleza, Brazil; 8 Erasmus MC, University Medical Center, Rotterdam, The Netherlands

**Keywords:** Leprosy, Post-exposure prophylaxis, Rifampicin, Clarithromycin, Blanket campaigns, High-endemic areas

## Abstract

**Background:**

Leprosy is an infectious disease with a slow decline in global annual caseload in the past two decades. Active case finding and post-exposure prophylaxis (PEP) with a single dose of rifampicin (SDR) are recommended by the World Health Organization as measures for leprosy elimination. However, more potent PEP regimens are needed to increase the effect in groups highest at risk (i.e., household members and blood relatives, especially of multibacillary patients). The PEP++ trial will assess the effectiveness of an enhanced preventive regimen against leprosy in high-endemic districts in India, Brazil, Bangladesh, and Nepal compared with SDR-PEP.

**Methods:**

The PEP++ study is a cluster-randomised controlled trial in selected districts of India, Brazil, Bangladesh, and Nepal. Sub-districts will be allocated randomly to the intervention and control arms. Leprosy patients detected from 2015 − 22 living in the districts will be approached to list their close contacts for enrolment in the study. All consenting participants will be screened for signs and symptoms of leprosy and tuberculosis (TB). In the intervention arm, eligible contacts receive the enhanced PEP++ regimen with three doses of rifampicin (150 − 600 mg) and clarithromycin (150 − 500 mg) administered at four-weekly intervals, whereas those in the control arm receive SDR-PEP. Follow-up screening for leprosy will be done for each individual two years after the final dose is administered. Cox’ proportion hazards analysis and Poisson regression will be used to compare the incidence rate ratios between the intervention and control areas as the primary study outcome.

**Discussion:**

Past studies have shown that the level of SDR-PEP effectiveness is not uniform across contexts or in relation to leprosy patients. To address this, a number of recent trials are seeking to strengthen PEP regimens either through the use of new medications or by increasing the dosage of the existing ones. However, few studies focus on the impact of multiple doses of chemoprophylaxis using a combination of antibiotics. The PEP++ trial will investigate effectiveness of both an enhanced regimen and use geospatial analysis for PEP administration in the study communities.

**Trial registration:**

NL7022 on the Dutch Trial Register on April 12, 2018. Protocol version 9.0 updated on 18 August 2022 https://www.onderzoekmetmensen.nl/en/trial/23060

**Supplementary Information:**

The online version contains supplementary material available at 10.1186/s12879-024-09125-2.

## Background

Leprosy, or Hansen’s disease, is an infectious disease caused by *Mycobacterium leprae* [1]. Although the transmission is not fully understood, the main mode is human-to-human via respiratory aerosols through coughing and sneezing. Prolonged and frequent close contact with an infectious person who has not started treatment is necessary for transmission [1, 2]. Therefore, individuals living in the same household as a person affected by the disease, neighbours and other family members are at a higher risk of being infected [2].

In 2022, the number of new leprosy patients reported globally was 174,087. The majority of the world’s leprosy patients (78%) live in three countries: India (60%), Brazil (11%) and Indonesia (7%). An additional 17% come from the next 20 global priority countries, including Bangladesh and Nepal [3]. Between 2010 and 2019, the annual decrease in new leprosy cases globally was a modest 2% per year [4]. Therefore, new interventions, such as more precise active case finding and chemoprophylaxis, are essential to reduce the global caseload.

Trials to study the effectiveness of chemoprophylaxis to prevent leprosy have been conducted for decades. The first successful leprosy chemoprophylaxis trials were conducted with dapsone administered weekly or biweekly for months or years with limited effectiveness [5]. More research was required for more powerful single dose regimens. Rifampicin (RMP), recognised as the most effective bactericidal agent against *M*. *leprae*, was subsequently researched [6]. A single dose of rifampicin as leprosy post-exposure prophylaxis (SDR-PEP) was administered to close contacts of leprosy patients to prevent the disease in several studies [7–9].

The effectiveness of SDR-PEP as chemoprophylaxis was most firmly established in COLEP, a randomised, placebo-controlled, double-blind trial in Bangladesh [7]. The COLEP study found an overall reduction in leprosy incidence of 57% among contacts in the intervention group in the first two years [10]. Feasibility and acceptability of implementing SDR-PEP in routine leprosy control programmes was investigated thereafter in eight countries in the leprosy post-exposure prophylaxis (LPEP) programme [8, 11]. As a result of these successful studies, the screening of close contacts of new leprosy patients combined with administration of SDR-PEP has been included in the World Health Organization (WHO) Guidelines for the Diagnosis, Treatment and Prevention of Leprosy [12]. Despite the overall protective effect of 57%, it was much lower among blood-related contacts and household members of multibacillary (MB) leprosy cases [10]. Therefore, a more potent post-exposure prophylaxis (PEP) regimen is needed to prevent disease especially among those at greater risk of developing the disease. 

In 2016, NLR developed the idea for a large multi-country trial testing the effectiveness of an enhanced PEP regimen to significantly reduce the new case detection and stop the transmission of leprosy. An international expert meeting recommended a regimen consisting of three doses of a combination of two highly bactericidal and accessible antibiotics: RMP and moxifloxacin (MXF) for adults and RMP and clarithromycin (CLR) for children [13]. In 2018, however, the European Medicines Agency (EMA) restricted the use of MXF as preventive treatment because of potential long-lasting and disabling side effects. In response to these restrictions, leprosy experts recommended to use the combination of RMP and CLR for both adults and children. This combination therapy using repeated doses has been tested in a nude mouse model [14]. Results showed that the PEP++ combination (RMP/CLR) has a greater effect compared to any single antibiotic. This increased effectiveness has not, however, been tested in human populations in endemic countries to date. In this trial, we hypothesise that the leprosy incidence will be reduced more substantially in areas where the enhanced regimen is administered, thus demonstrating the effectiveness of the enhanced regimen.

## Methods and design

### Objectives

The primary objective of the PEP++ randomised controlled trial (RCT) is to provide evidence of the effectiveness of an enhanced post-exposure prophylaxis regimen (PEP++) compared to the currently recommended regimen of SDR-PEP. Each study district will have an adverse events committee to monitor the frequency and severity of such events in each study arm and ensure participant safety.

Secondary study objectives seek to provide evidence of:

- The acceptability and cost-effectiveness of PEP++ as a preventive intervention
- The accuracy of geospatial methods to identify target areas for blanket campaigns, and
- The effectiveness of community education and behaviour change (CEBC) interventions to change the perception of leprosy and reduce stigma

### Study design

The study will use a cluster-randomised non-blinded controlled trial design with two arms to compare the effectiveness of three doses of RMP/CLR (the PEP++ regimen) with SDR-PEP in the prevention of leprosy disease among contacts of newly diagnosed leprosy patients. The randomisation units will sub-district divisions in each country context that will be randomly allocated to the two study arms.

All eligible leprosy patients in the districts will be asked to enumerate their close contacts. These individuals will subsequently be approached and enrolled in the study. Those contacts who provide informed consent are screened for signs of leprosy and tuberculosis and for inclusion eligibility. Contacts of leprosy patients living in the intervention arm receive the PEP++ regimen while those in the control arm receive SDR-PEP. All participants included in the study will be followed up two years after receiving the final dose of PEP++ or SDR-PEP.

**Fig. 1:**
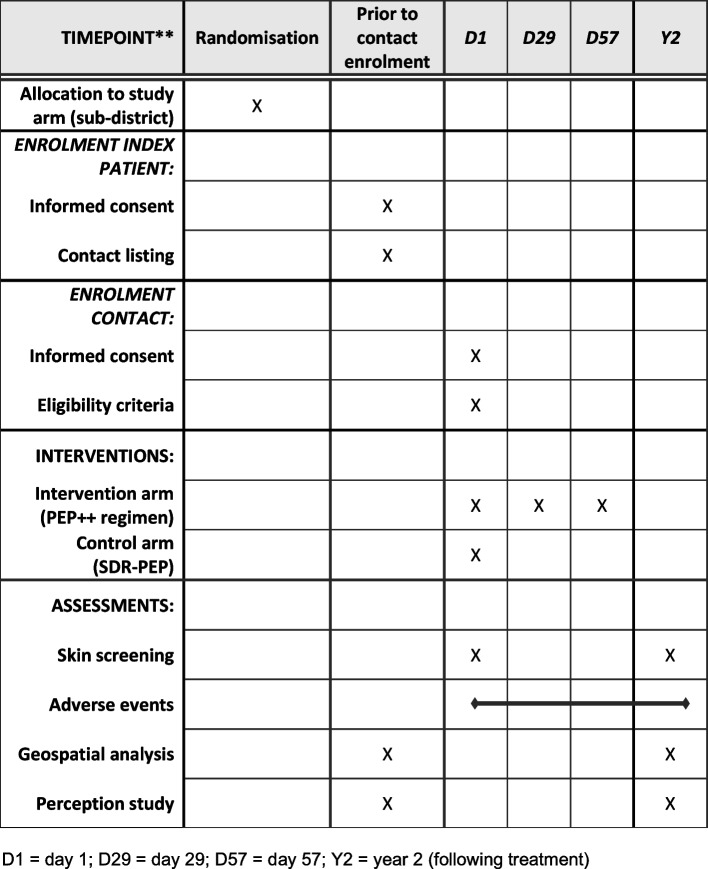
Study schedule of enrolment, interventions, and assessments

The residences of the leprosy cases approached for the study will be geocoded in order to develop epidemiological maps that identify clusters of leprosy cases. After the regimen trial intake is concluded, blanket campaigns with SDR-PEP will be implemented in these clusters (high-risk areas) to reduce leprosy incidence at the population level. The study schedule is presented in Fig. 1. A checklist of Recommendations for Clinical Intervention Trials (SPIRIT) is also available (Supplementary file 1).

### Study setting

This study will be conducted in two districts in India, Brazil, and Bangladesh and three districts in Nepal. Together these four countries accounted for 128,727 new leprosy cases in 2022, or 73.9% of the global caseload of 174,087 [3]. The districts/municipalities were selected per country based on the number of new leprosy cases detected in recent years, availability of contact screening and diagnostic services for leprosy, and logistical feasibility. For ease of study implementation and operational considerations, the districts are located in a single state or province in each country. A mix of urban, rural, and peri-urban settings was sought in order to show the replicability of the intervention across contexts in the future.

### Participants

In this trial, we approach recently detected leprosy patients (index cases) as derived from leprosy programme registries in the four countries. The total number of new leprosy cases per study site is presented in Table 1. These patients must have been detected in 2015 or later, give informed consent to participate in the study, and be willing to list their close contacts. The target population for preventive treatment is comprised of household contacts, family members, neighbours, and other social contacts (jointly denominated as ‘close contacts’) as listed by the index cases. These contacts are individuals who have had intensive contact with a leprosy patient for approximately 20 h per week during at least three months in the year before the index case was diagnosed. From previous studies, it is expected that approximately 20 close contacts per index case will be listed, depending on the study setting. All close contacts listed by an index case and located by the research staff are enrolled in the study with a unique identification code (UIC) and receive information on the study.

**Table 1:** Total number of new leprosy cases per study site

District/Municipality	Total number of new leprosy cases (2015–2020)
**India**^**a**^	
Chandauli	1,700
Fatehpur	2,329
**Total India**	**4,029**
**Brazil**	
Fortaleza	3,580
Sobral	498
**Total Brazil**	**4,078**
**Nepal**	
Dhanusha	1,724
Mahottari	885
Sarlahi^b^	411
**Total Nepal**	**3,020**
**Bangladesh**	
Rangpur	1,734
Nilphamari	1,671
**Total Bangladesh**	**3,405**
**Study total**	**14,532**

Sources – National Health Information System on Notifiable Diseases (SINAN; Brazil); Primary Care Health Centres at sub-district level and district consolidation (India, Nepal, and Bangladesh)

^a^Fiscal year is from April 2015 to March 2021

^b^Sarlahi coverage is partial (9 out of 20 municipalities) due to SDR-PEP roll-out in other areas

The contacts who consent to participate in the trial will first be screened by the research staff for signs and symptoms of leprosy and tuberculosis (TB) and checked for eligibility criteria. Exclusion criteria for the administration of preventive treatment are refusal to provide informed consent, a history of liver, renal or cardiac disease or a known allergy to RMP and/or CLR. Contacts are also temporarily ineligible if: pregnant or breastfeeding, under the age of two years, received RMP for any reason in the last two years or using contraindicated medicines for non-chronic use. If these conditions change during the trial intake period, they may still be included in the study. In the presence of any possible signs of leprosy, the participant will be referred to the closest qualified health centre for confirmation of diagnosis. Those who are confirmed as a new leprosy case are subsequently requested to list their close contacts for enrolment in the study.

### Randomisation

The allocation of intervention and control arms in the countries has been done blindly using stratified randomisation. For each country, the randomisation unit has been determined via consultation with local government officials and project staff. This resulted in the use of territories/neighbourhoods in Brazil, blocks in India, municipalities in Nepal and unions in Bangladesh. These randomisation units were divided into two strata based on the number of clusters and the number of cases in clusters (based on geospatial analysis results with data from 2015–2020) followed by random sampling into intervention or control arms.

### Sample size

The calculation of the sample size is based on the primary objective to find differences in new case detection rates (NCDR). The first is a difference in NCDR in the population in the intervention area after four years, compared to the baseline rate in 2019. The year 2019 is selected as the baseline to avoid any effect of the COVID-19 pandemic. The second is the difference in incidence rates in the contact groups between the close contacts who have received PEP++ and controls who have received SDR-PEP.

The sample size calculation for the difference in rates in the close contacts is based on the NCDR found in contacts in the COLEP trial sample. The NCDR in the SDR intervention group was 291/100,000 over 2 years, or an annual rate of 146/100,000. To reduce this rate by 50%, using a power of 90%, a significance level of 0.05, design effect of 1.5, correction for non-eligibility of 25% and loss to follow-up of 25%, we aimed to enrol 202,360 close contacts. This should result in giving PEP to at least 162,000 participants.

### Outcome measures

The primary outcome measures of the trial will be the number of new leprosy cases detected, the number of child cases detected, and the incidence rate ratios of each arm compared with 2019 baseline. These will be measured at the two-year follow-up of the trial. In addition, the cost-effectiveness and acceptability of PEP++ as a preventive intervention will be measured as separate side studies. The added value of geospatial methods will be measured by the proportion of new leprosy cases in clusters and non-clusters.

### Intervention implementation

Post-exposure prophylaxis will be offered to all eligible participants. Those in the intervention arm will receive three doses of RMP/CLR (PEP++ regimen) with four-week intervals. To increase the acceptability and reduce the potential adverse events, extended-release clarithromycin will be offered where available for purchase. An additional four-week tolerance exists for each repeated dose so that an interval of eight weeks between doses is valid. Participants in the control arm will receive SDR-PEP. The medication dosages of the PEP++ and SDR-PEP regimens per age group are presented in Table 2. Both regimens will be provided only under supervision of the research staff and/or medical officers. The date, dosage and type of PEP will be recorded for each eligible person in the study.

**Table 2:** PEP +  + regimen dosage by age group

	**PEP++ regimen**^**a**^	**SDR-PEP**
≥ 15 years	600 mg RMP + 500 mg CLR	600 mg RMP
13–14 years	450 mg RMP + 500 mg CLR	450 mg RMP
10–12 years	450 mg RMP + 450 mg CLR^b^	450 mg RMP
6–9 years	300 mg RMP + 300 mg CLR^b^	300 mg RMP
2–5 years	150 mg RMP^b^ + 150 mg CLR^b^	150 mg RMP^b^

^a^A four-weekly dose at day 1, 29 and 57 with four-week tolerance (up to 56 days between doses)

^b^ml equivalent in paediatric suspension or capsules, depending on availability

The risk of inducing rifampicin resistance in *M. leprae* or *M. tuberculosis* after providing a single dose of rifampicin is considered negligible because only repeated doses of a single antibiotic will increase the risk of resistance [15]. Moreover, participants that have received rifampicin in the last two years or that show any signs or symptoms of TB or leprosy will not receive PEP. Contacts confirmed to have TB or leprosy will be treated according to the national guidelines.

### Leprosy perception

A person’s perception of leprosy can negatively affect health-seeking behaviour and the acceptance of new interventions [16]. Therefore, contextualised community education and behaviour change (CEBC) materials are developed as part of the study to change the perception (knowledge, attitudes, beliefs, and emotions) regarding leprosy and reduce stigma, as well as to increase the community acceptance of preventive treatment. First, a leprosy perception study was conducted in each country to investigate the perceptions of leprosy patients, contacts, community members and health workers regarding leprosy, i.e., the way people see leprosy, what they know about leprosy and their attitudes, beliefs and reported behaviour towards persons affected by leprosy [17, 18]. A mixed-method approach will be used to measure the perception, including in-depth interviews, focus group discussions (FGDs), the knowledge, attitudes, and practices (KAP) tool, the Explanatory Model Interview Catalogue Community Stigma Scale (EMIC-CSS) and the Social Distance Scale (SDS). During a workshop, leprosy affected persons and other key stakeholders will use the outcomes of the perception study to develop messages for the CEBC materials. The CEBC materials will be distributed throughout the implementation areas and piloted before the start of the trial [19].

### Geospatial methods

Prior to the trial implementation, geospatial analysis was done to develop epidemiological maps and determine the target areas for blanket campaigns. The latitude and longitude of all patient’s residents registered from 2015 to 2021 were collected using the mobile application MapitPro (version 7.6.0). All data points were checked for clustering in open-source Quantum Geographic Information System (QGIS) version 3.4.1 (QGIS Developer team, Madeira (2018)). A contextualised spatial analysis approach was developed to identify small and precise clusters in each implementation area. This approach included non-statistical geospatial methods combined with expert consultation. During the expert consultations, country-specific definitions of a cluster were determined considering the local context [20].

### Blanket campaigns

As predicted by mathematical modelling, a larger reduction in new cases detected can be achieved by implementing additional blanket campaigns in the identified clusters. For each index case in a cluster, the households not listed as close (neighbour) contacts within a radius of approximately 100 m will be visited until 80 to 100 participants (blanket contacts) are included in the study. If this results in over 80% of the cluster area being covered, then the entire population will be invited for participation. These blanket contacts are, similar to the close contacts, asked for consent to participate in the trial, screened for signs and symptoms of leprosy and when eligible, are provided SDR-PEP.

### Data collection

Data collection will be done offline using the Research Electronic Data Capture (REDCap) system created at Vanderbilt University in the United States. Specific forms and modules were developed by the study teams, translated to the local languages, and downloaded to tablets and smartphones for field usage. First, a unique identification code (UIC) will be created for each close contact in the trial to anonymise the data. The UIC is linked to the index case and will be used to link the different data collection forms that will be created for each contact. Contacts are then visited at home and asked to sign a consent form if they are willing to participate. Disclosure of the identity of the index case is avoided whenever possible when approaching neighbours and social contacts.

From all contacts, we will collect demographic data (e.g., name, age, gender), information on the relationship with the index case and the results from the screening and eligibility questions in the close contact registration form. The date and dosage of the PEP administered is recorded in either the SDR-PEP form or PEP++ forms (i.e., first, second and third dose). Moreover, any side effects or adverse events due to SDR-PEP or PEP++ will be registered in the adverse event form. During the trial, the GPS coordinates of all participants and new leprosy patients will be collected to determine the spatial effectiveness of PEP. all case records and GPS data are stored temporarily on the mobile study devices and uploaded daily to a national server in each country.

### Data analysis

Data from the PEP++ trial will be analysed primarily through quantitative methods using descriptive analysis for all variables. Table 3 outlines the tools to be used to measure and analyse the study outcomes.

**Table 3:** PEP++ project outcomes and statistical analysis plan

Objective	Outcome	Hypothesis	Outcome measure	Method of analysis
To provide evidence of the effectiveness of an enhanced post-exposure prophylaxis regimen (PEP++) compared to SDR-PEP	Primary: contacts diagnosed with leprosy	PEP++ offers a higher protection against leprosy disease manifestation than SDR-PEP	Number of persons with leprosy disease detected at 24 months after receiving prophylaxis	Descriptive statistics; Cox’ proportion hazards analysis and Poisson regression comparing rates between the intervention and control areas
To show the cost- benefit and acceptability of the enhanced chemoprophylaxis regimen	Secondary: Cost–benefit of the enhanced regimen compared with effectiveness of SDR-PEP	PEP++ is a cost-effective strategy in leprosy control and the increased effectiveness of the intervention will justify additional costs from multiple doses	Number of contacts screened, cases prevented, and disabilities avoided against operational costs and out-of-pocket expenses	Health economic evaluation
	Secondary: Acceptability of the enhanced regimen compared with SDR-PEP	The new regimen will be accepted by contacts and health professionals	Proportion of eligible close contacts who take all three doses; qualitative interview results indicating that PEP++ is acceptable	Descriptive statistics of contacts who took medication; qualitative content analysis of interviews; semi-structured interviews, FGDs
To provide evidence of increased transmission of *M. leprae* in cluster areas identified through GIS-based mapping compared to non-cluster areas	Secondary: Contacts diagnosed with leprosy in identified high-transmission clusters (2014–19 index cases)	Detection of new leprosy cases will be higher in the cluster areas than in non-cluster areas due to higher local transmission and relative risk	Rate ratios of all new leprosy cases and child cases in cluster areas as compared with non-cluster areas (as a result of intervention as well as passive cases); rate ratio	Poisson regression comparing rates between cluster and non-cluster areas
To demonstrate the effectiveness of the community education and behaviour change interventions to change the perception of leprosy in the study districts	Secondary: Levels of knowledge, attitudes, and stigma in each district	New, contextualised education materials and interventions will increase community knowledge regarding leprosy and reduce stigma so as to promote early detection	Change in KAP, EMIC-CSS and SDS scores and interviews/FGDs in all four target groups	Descriptive statistics; comparison of mean/median scores between baseline and end-line surveys; effect size; correlations between exposure to the interventions and KAP, EMIC-CSS and SDS scores; multivariate regression; qualitative content analysis of interviews, FGDs, and observations
To demonstrate proof of concept of the potential for the PEP++ approach to reduce, and ultimately stop, the transmission of *M. leprae* in a target area	Primary: Detection of new autochthonous child cases of leprosy	Reduction in new case and child case detection will be higher in intervention compared with control areas; Overall and child case detection levels will reduce against baseline (2019) detection	Rate ratio of new case and child case detection per 1,000,000 population; total number of new cases per district	Descriptive statistics, comparison of new case and child case detection rates between the intervention and control areas

### Dissemination

Study outcomes are expected to be applicable for wider replication and scaling up throughout the four study countries, as well as in other countries with highly endemic regions. The national and international study teams will write extensively on the outcomes of the study. All publications will go before a project publications committee with the approval of the local Principal Investigator before submission to open-access, peer-reviewed journals. It will also be well represented in international congresses and events by the study teams in the four countries involved. Communications with the WHO and relevant Ministries of Health will take place throughout the project with the long-term sustainability of the approach in mind.

## Discussion

Although SDR-PEP distribution to household contacts is currently the standard WHO-recommended preventive treatment for leprosy, it may not be sufficient to stop leprosy infection in contacts who are already incubating leprosy disease. In the COLEP trial, the effectiveness of SDR-PEP was lowest among blood-relatives and household contacts [10]. To meet the needs of this group, recent studies with stronger regimens and/or different antibiotics have been conducted or are ongoing. The PEOPLE project in the Comoros and Madagascar assessed the effectiveness of a single double dose of rifampicin (SDDR) among household contacts [21]. Preliminary study data show that SDDR is effective in reducing the risk of leprosy even among household contacts and also at the population level (Hasker et al., accepted for publication). However, the effectiveness was still limited and the authors suggested that other stronger regimens should be tested. Therefore, the same study group is currently evaluating the effectiveness of a new enhanced PEP regimen consisting of bedaquiline and rifampicin in a four-year trial in the Comoros [22]. Bedaquiline is a more potent and longer acting antibiotic, which is used for latent TB infection and multidrug resistant pulmonary TB. It has not been used as leprosy preventive treatment before and is therefore undergoing a series of drug trials as part of this study. Rifapentine is another potent and longer-acting antibiotic that has been known as a promising candidate in the fight against leprosy for many years. A recent study in Southwest China showed that a single dose of rifapentine reduced the cumulative incidence of leprosy among household contacts by 84% compared with an untreated control group. It was considerably more effective than single-dose rifampicin in the target group [23]. If made available at affordable rates in all leprosy endemic countries, these powerful bactericidal agents offer hope of better single-dose protective regimens in the future.

Nevertheless, the study by Lenz et al. (2020) of *M. leprae*-infected nude mice showed that four multi-drug, multi-dose regimens were more effective to stop bacterial growth than any single-dose intervention, even those using multiple antibiotics with rifapentine [14]. The team concluded that ‘multi-dose PEP may be required to control infection in highly susceptible individuals with subclinical leprosy to prevent disease and decrease transmission.’ The PEP++ trial seeks to provide evidence to back up these laboratory findings and show that an enhanced multi-dose regimen of existing antibiotics is a powerful tool to reduce the risk of leprosy across a range of health systems under real-life field conditions.

Finally, several studies show that targeting the households of leprosy patients only is insufficient to stop transmission [10, 21, 24]. Community members within a 100-m radius of the leprosy patient’s house are also at a higher risk to develop leprosy. Targeted population-wide approaches or focal mass drug administration campaigns are therefore also needed. Little evidence has been published on the impact and cost-effectiveness of these approaches. The five-islands study in Indonesia by Bakker et al. (2005) compared a population-wide SDR-PEP approach with a contact-based approach [6]. They found a larger decrease in the risk of leprosy on the island using the population-wide approach. However, this was an island setting with low mobility of the population. Therefore, to collect additional evidence on the effectiveness of population-wide approaches, we will conduct blanket campaigns in identified clusters targeting 80 to 100 community members per index case.

A large-scale reduction in transmission across a large area will require a comprehensive application of ‘PEP services’, including SDR-PEP distribution to neighbours and social contacts, active case detection, community education and capacity strengthening of health workers. Following the individual- and population-level data analysis of this study, we expect to have evidence that it is possible to accelerate the reduction in leprosy incidence within a few years through implementation of the enhanced PEP++ regimen combined with SDR-PEP blanket campaigns and health system strengthening components as appropriate.

### Supplementary Information

**Supplementary Material 1.**

## Data Availability

No datasets were generated or analysed during the current study.

## Abbreviations

CEBC: Community education and behaviour change
CLR: Clarithromycin
COLEP: Contact transmission and chemoprophylaxis in leprosy study
EMA: European Medicines Agency
EMIC-CSS: Explanatory Model Interview Catalogue Community Stigma Scale
GIS: Geographic Information System
GPS: Global Positioning System
KAP: Knowledge, Attitudes and Practices measure
LPEP: Leprosy post-exposure prophylaxis programme
MDT: Multi-drug therapy
PEP: Post-exposure prophylaxis
PEP++: Enhanced multi-drug, multi-dose regimen leprosy post-exposure prophylaxis
QGIS: Quantum Geographic Information System
RCT: Randomised controlled trial
REDCap: Research Electronic Data Capture System
RMP: Rifampicin
SDR-PEP: Single dose of rifampicin as leprosy post-exposure prophylaxis
SDS: Social Distance Scale
SIMCOLEP: Stochastic individual-based model for transmission and control of leprosy
TB: Tuberculosis
UIC: Unique identification code
WHO: World Health Organization
